# The RXFP2-PLC/PKC signaling pathway mediates INSL3-induced regulation of the proliferation, migration and apoptosis of mouse gubernacular cells

**DOI:** 10.1186/s11658-023-00433-0

**Published:** 2023-02-27

**Authors:** Shouxing Duan, Xuewu Jiang, Jianhong Li, Maxian Fu, Zhuo Li, Yiyi Cheng, Yangmu Zhuang, Ming Yang, Wenfeng Xiao, Hongyan Ping, Yao Xie, Xiaojun Xie, Xuan Zhang

**Affiliations:** 1 Department of Pediatric Surgery, Huazhong University of Science and Technology Union Shenzhen Hospital (Nanshan Hospital), No. 89 Taoyuan Road, Shenzhen, 518052 Guangdong China; 2 Department of Pediatric Surgery, The First Affiliated Hospital of Shantou University Medical College, No. 57 Changping Road, Shantou, 515041 Guangdong China; 3 Department of Pediatric Surgery, Pingshan District Maternal and Child Healthcare Hospital of Shenzhen, Pingshan General Hospital of Southern Medical University, No. 6 Longxingnan Road, Shenzhen, 518118 Guangdong China; 4 Department of Pediatric Surgery, The Second Affiliated Hospital of Shantou University Medical College, No. 69 Dongxiabei Road, Shantou, 515041 Guangdong China; 5 Department of Radiology, Cancer Hospital of Shantou University Medical College, No. 7 Raoping Road, Shantou, 515041 Guangdong China; 6 Department of General Surgery, The First Affiliated Hospital of Shantou University Medical College, No. 57 Changping Road, Shantou, 515041 Guangdong China

**Keywords:** INSL3, RXFP2, PLC/PKC, Gubernaculum, Signaling pathway

## Abstract

**Background:**

Testicular hypoplasia can affect the sexual and reproductive ability in adulthood, and even increase the risk of cancer. Abnormal development of the gubernaculum is one of the important factors of testicular hypoplasia. Therefore, a study of the structure and function of the gubernaculum is an important but neglected new breakthrough point for investigating the normal/abnormal development of the testis. Previous findings showed that Insulin like factor 3 (INSL3) is a key factor regulating the growth of gubernaculum, however, the mechanism by which INSL3 acts on the gubernaculum remains unknown. Therefore, we probed the mechanism associated with INSL3-induced the proliferation, migration, and apoptosis of gubernacular cells in mice.

**Methods:**

A culture cell model of neonatal mice gubernaculum is established by INSL3 intervention. We blocked PLC/PKC signaling pathway with U73122 pretreat to investigate the role of the PLC/PKC signaling pathway. The changes of cell proliferation, migration, and apoptosis were detected by molecular biological methods. In addition, the levels of PCNA and F-action were detected by immunofluorescence and western blotting.

**Results:**

We found that INSL3 can promote the proliferation and migration of gubernacular cells and inhibit their apoptosis, meanwhile, INSL3 significantly up-regulated PLC/PKC protein phosphorylation. However, treatment with the PLC/PKC signaling pathway inhibitor U73122 significantly inhibited these effects of INSL3. Besides, we found that INSL3 could up-regulate the protein expression level of PCNA and F-actin, while the PCNA and F-actin expression was significantly weakened after U73122 pretreatment.

**Conclusions:**

This research revealed that INSL3 binding to RXFP2 may up-regulate the expression levels of PCNA and F-actin by activating the PLC/PKC signaling pathway to promote the proliferation and migration of gubernacular cells. It suggests that the RXFP2-PLC/PKC axis may serve as a novel molecular mechanism by which INSL3 regulates growth of the gubernaculum.

**Supplementary Information:**

The online version contains supplementary material available at 10.1186/s11658-023-00433-0.

## Background

The testis is the main organ of the male reproductive system, which affects the function of the whole male reproductive system. Testicular hypoplasia can affect the sexual and reproductive ability in adulthood, and even increase the risk of cancer [1, 2]. Thus, it is very important to study the influencing factors of testicular descent [3].

Studies have shown that abnormal myoblast differentiation in the gubernaculum is related to failure of testicular descent. Abnormal development of the gubernaculum is one of the important factors of cryptorchidism [4, 5]. Many clinical observations have also confirmed the correlation between the developmental stage of testis and gubernaculum [6]. In the transabdominal stage of testicular descent, the testicle descends into the inguinal region, with rapid proliferation of gubernacular cells, which is accompanied by degeneration of the cranial suspensory ligament. As the gubernaculum degrades, the testis descends from the bottom of the abdominal cavity to the bottom of the scrotum, completing the inguinal scrotal stage of testicular descent [7]. Therefore, a study of the structure and function of the gubernaculum is an important but neglected new breakthrough point for investigating the normal/abnormal development of the testis, and even the male reproductive system.

Insulin like factor 3 (INSL3) is considered to be a key factor affecting the transabdominal descent of the testis, and it regulates the descent of the testis by influencing the growth and development of the gubernaculum [8, 9]. Studies have shown that INSL3 binds to its specific receptor RXFP2 (Relaxin family peptide receptor 2) located in the gubernaculum, stimulating the proliferation of gubernaculum, and controlling the growth and development of gubernaculum [8, 10–12]. However, the mechanism by which INSL3 acts on the gubernaculum is still not clear.

The specific receptor RXFP2 belongs to the G protein-coupled receptor (GPCR) family and plays a biological role by mediating its downstream signaling pathway [13, 14]. Many studies have confirmed that the protein kinase C (PKC) signaling pathway plays an important role in intercellular signal transport, transcriptional regulation of cell function, opening/closing of an ion channel, and energy metabolism, and it can regulate cell proliferation, differentiation, contraction, secretion and metabolism [15–18]. In a previous study on the effect of diethylbestrol (DES) on mouse gubernacular cells, it was found that DES regulated the proliferation of mouse gubernacular cells through the PLC‐Ca^2+^‐CREB pathway [19]. These data suggest that the PLC/PKC pathway may mediate biological functions in gubernacular cells. However, none of the studies have confirmed that the PLC/PKC signaling pathway mediates the biological process through which INSL3 regulates the proliferation, migration, and apoptosis of gubernacular cells.

A schematic diagram to illustrate the experimental system for function confirmation of INSL3 regulating gubernacular cells and signaling pathways analysis is shown in Additional file 1: Fig. S1. In this study, we showed that INSL3 plays an important role in growth of the gubernaculum, which can promote the proliferation and migration of gubernacular cells and inhibit their apoptosis. Interestingly, U73122 was added to suppress the PLC/PKC signaling pathway, and the above-described regulatory function of INSL3 in gubernacular cells was significantly reduced. This suggests that the PLC/PKC signaling pathway is also involved in these biological processes. Therefore, the RXFP2-PLC/PKC axis may serve as an alternative molecular mechanism by which INSL3 regulates growth of the gubernaculum.

## Materials and methods

### Animals and ethics

Kunming mice, 8–10 weeks of age and free of murine specific pathogens, were purchased and maintained at the Animal Research Laboratory in the Medical College of Shantou University. All animals received excellent nutrition, were drug-free, and exhibited normal mental and neurological states. The Shantou University Medical College Animal Experimental Ethics Committee approved the use of Kunming mice for protocol purposes (NO. SUMC 2018-064).

### Cell culture and experimental grouping

Three-day-old neonatal mice were euthanized by removing the cervical vertebra and gubernaculum tissue was removed immediately using a surgical magnifying glass. The tissue was digested in Dulbecco’s modified Eagle’s medium (DMEM) containing Type I collagenase (1 mg/ml) at 37 °C for 1 h, and then diluted in a 3- to fourfold higher volume of medium without estrogen. The medium was decanted, filtered through a 25-μm filter, centrifuged at 1000 rpm for 5 min (centrifugation radius 15 cm), and the supernatant was then discarded. The cell pellet was added to DMEM containing charcoal-stripped FBS (CS-FBS) (Invitrogen, USA) but lacking estrogen (10% v/v), aerated to create a single-cell suspension, seeded into a 25-ml culture flask, and incubated in a 5% CO 2 incubator at 37 °C with saturated humidity [20, 21]. Cells were randomized into groups consisting of control group, INSL3 group (cells treated by 0.02 ng/ml INSL3), U73122 (Phospholipase C (PLC) inhibitor) group (cells pretreated by 1 μmol/L U73122) and INSL3 + U73122 group (cells pretreated by 1 μmol/L U73122 for 10 min, then treated by 0.02 ng/ml INSL3) [19, 20]. The gubernacular cells were treated with INSL3 for 48 h, and the mouse INSL3 was from Phoenix Pharmaceutics.

### Hematoxylin–eosin staining and cell counting

Gubernaculum cells were harvested for hematoxylin and eosin (HE) staining, and the cell morphology was observed under a phase contrast microscope (200 ×, Leica, Germany). The cells in suspension were counted at the time of subculture. A small amount of uniform cell suspension was dropped into the gap between the counting plate and cover slip. Cells in the four regions were counted, and those on the line were counted only when they were in the left upper corner, not in the right lower corner (Additional file 2: Fig. S2). The cell concentration was calculated using the following equation: (number of cells/ml) = (total number of cells in four regions) × 10,000 × dilution fold.

### CCK8 assay

Gubernacular cells were seeded at a density of 3.5 × 10^3^ cells in each well of the 96-well plates and cultured for 12 h in DMEM containing 5% CS-FBS (Invitrogen, USA). Cell viability was detected using the CCK-8 assay with cell counting kit-8 (Dojindo, Mashikimachi, Japan) following the manufacturer’s instructions. Absorbance values at 450 nm were measured using an automatic multiwell spectrophotometer.

### Immunofluorescence analysis

The cells were fixed with 4% paraformaldehyde for 15 min, washed three times with PBS, permeabilizaed in 0.2% Triton X-100 in PBS for 10 min, and blocked with 1% BSA. Primary antibodies against PCNA (1:200, AF0239, Affinity, USA) at 4 °C overnight. Fluorescein isothiocyanate (FITC)-conjugated rabbit antigoat IgG secondary antibody (1:500, BA1110, Boster, China) was added for 1 h at room temperature. F-actin was detected by incubation with 5 μg/ml FITC-phalloidin (1:10, P5282, Sigma, USA) in PBS for 40 min. The cover slip was incubated at room temperature for 60 min, sealed with glycerin, and visualized by a fluorescence microscopy (400 ×, Leica, Germany).

### Cell scratch assay

The cells were cultivated in ibidi dishes with culture inserts at the bottom. The inserts were removed to produce uniform scratches 500 μm wide when the cells were adherent exceeded 95% density. Cell debris was removed by pre-warmed PBS and then the cells were cultured in the incubator. The cell images were photographed at 0 h, 12 h and 24 h, respectively. The Image was analyzed by Image J software to calculate the scratch healing rate.

### Transwell assay

Transwell chambers with a polycarbonate membrane with 5 μm pores (Corning, USA) were coated with Matrigel basement membrane matrix (Corning, USA). Then, 5 × 10^5^ cells suspended in FBS-free medium were seeded into the upper chambers, and 600 µl medium containing 10% CS-FBS (Invitrogen, USA) was placed into the lower chambers. 24 h later, the medium was discarded, and the cells were fixed with 4% paraformaldehyde for 30 min at room temperature. 1% crystal violet was used to stain cells for 20 min. The cells invading the lower chamber were scanned and counted.

### TUNEL staining

In order to detect apoptosis, TUNEL staining was performed with an in situ apoptosis detection kit (KeyGen BioTECH, China). TUNEL analysis was performed according to the kit’s instructions. The cells were sealed and photographed under a fluorescence microscope (200 ×, Leica, Germany).

### Flow cytometry assay (FCA)

The degree of cell apoptosis was detected using the Annexin V-FITC Apoptosis Detection Kit (KeyGen BioTECH, China) according to manufacturer’s instructions. Specifically, cells were washed twice with PBS, and then 1 × 106 cells /ml suspension was prepared with 1 × Binding Buffer. Falcon test tube was filled with 100 μl cell suspension. Each sample was lightly mixed with 5 μl Annexin V and PI staining solution and placed at room temperature (20–25 °C) away from light for 15 min. 1 × Binding Buffer 400 μl was added to each test tube. Results were detected by flow cytometry (BD, USA) within 1 h.

### Western blot analysis

Total protein was extracted using Radioimmunoprecipitation Assay (RIPA) lysis buffer. After quantification of total protein using BCA protein assay kit (P0010S, Beyotime, China), protein samples were loaded onto sodium dodecyl sulfate polyacrylamide gel electrophoresis (SDS-PAGE), and then transferred onto PVDF membranes. The membrane was blocked with skim milk, followed by incubation with primary antibodies against PCNA (1:1000, AF0239, Affinity, USA), F-actin (1:1000, ab205, Abcam, UK), PLC (1:1000, ab302940, Abcam, UK), P-PLC (1:1000, ab76031, Abcam, UK), PKC (1:1000, ab181558, Abcam, UK), P-PKC (1:1000, ab109539, Abcam, UK) and GAPDH (1:5000, ab8245, Abcam, UK) at 4 °C overnight and then with horseradish peroxidase-conjugated secondary antibodies (1:1000, Abcam, UK) for 1 h at room temperature. After chemiluminescent reaction, the gel results were scanned and photographed with a gel imaging system, and the OD of the target band was analyzed using ImagePro software.

### Statistical analysis

All data are expressed as the mean ± standard deviation (SD). SPSS 22.0 software was used to analyze the data. The constituent ratio was determined by the Chi-square test, and multiple comparisons between groups were analyzed by the homogeneity test of variances and one-way analysis of variance (ANOVA). Pairwise comparison was analyzed from two independent samples using the Student’s t-test. P < 0.05 was considered as statistically significant.

## Result

### INSL3 promoted the proliferation of gubernacular cells, and U73122 inhibited the proliferative effect of INSL3

**Fig. 1:**
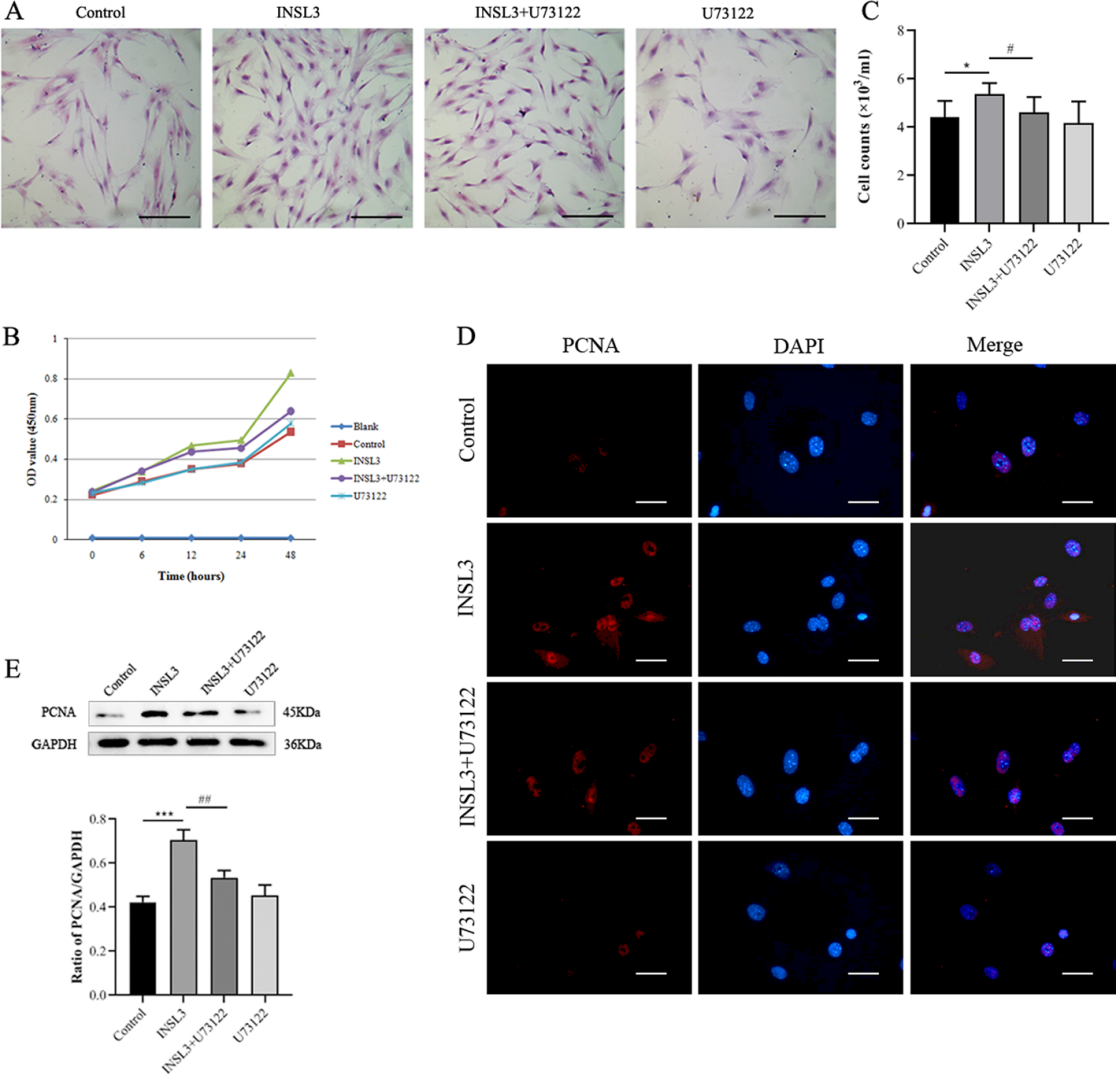
U73122 inhibited the effect of INSL3 on the proliferation of gubernacular cells. **A** Cell morphology was observed by HE staining. Scale bars, 50 μm. **B** Cell proliferation was detected by CCK8. **C** Cell counting measures changes in the number of cells. The counting method is shown in Additional file 2: Fig. S2. **D** The expression of proliferating cell nuclear antigen (PCNA) was observed by immunofluorescence. Scale bars, 20 μm.** E** The expression of PCNA protein was analyzed by Western blot. Data are mean ± standard deviation of three independent experiments. *^&#^P < 0.05, **^&##^P < 0.01, and ***^&###^P < 0.001

The results of HE staining and cell count showed that most of the cells were fibroblast-like, radial, or flame-like in shape, and the cell body was spindle or irregular triangle in shape, with good uniformity of cell morphology (Fig. 1A). In addition, the cell counting kit-8 (CCK-8) method was used to detect cell proliferation at 6 h, 12 h, 24 h, and 48 h, respectively. The results showed that there was a significant difference in cell proliferation after 48 h of culture, and INSL3 significantly promoted cell proliferation. Compared with the control group, cell proliferation was significantly increased in the INSL3 group (P < 0.05). U73122 inhibited the INSL3-induced cell proliferation (P < 0.05) (Fig. 1B). The results obtained by cell counting were consistent with those obtained by CCK-8 (Fig. 1C, Additional file 3: Table S1). Subsequently, we analyzed the expression of the proliferating cell nuclear antigen (PCNA) protein using immunofluorescence staining and Western blot. The results showed that PCNA expression was significantly increased in the INSL3 group (P < 0.001) compared with the control group, and U73122 inhibited the increase in PCNA expression caused by INSL3 (P < 0.01) (Fig. 1D, E). These results indicated that INSL3 promoted the proliferation of gubernacular cells, and U73122 inhibited the proliferative effect of INSL3.

### INSL3 increased the migration ability of gubernacular cells, and U73122 inhibited the pro-migration effect of INSL3

**Fig. 2:**
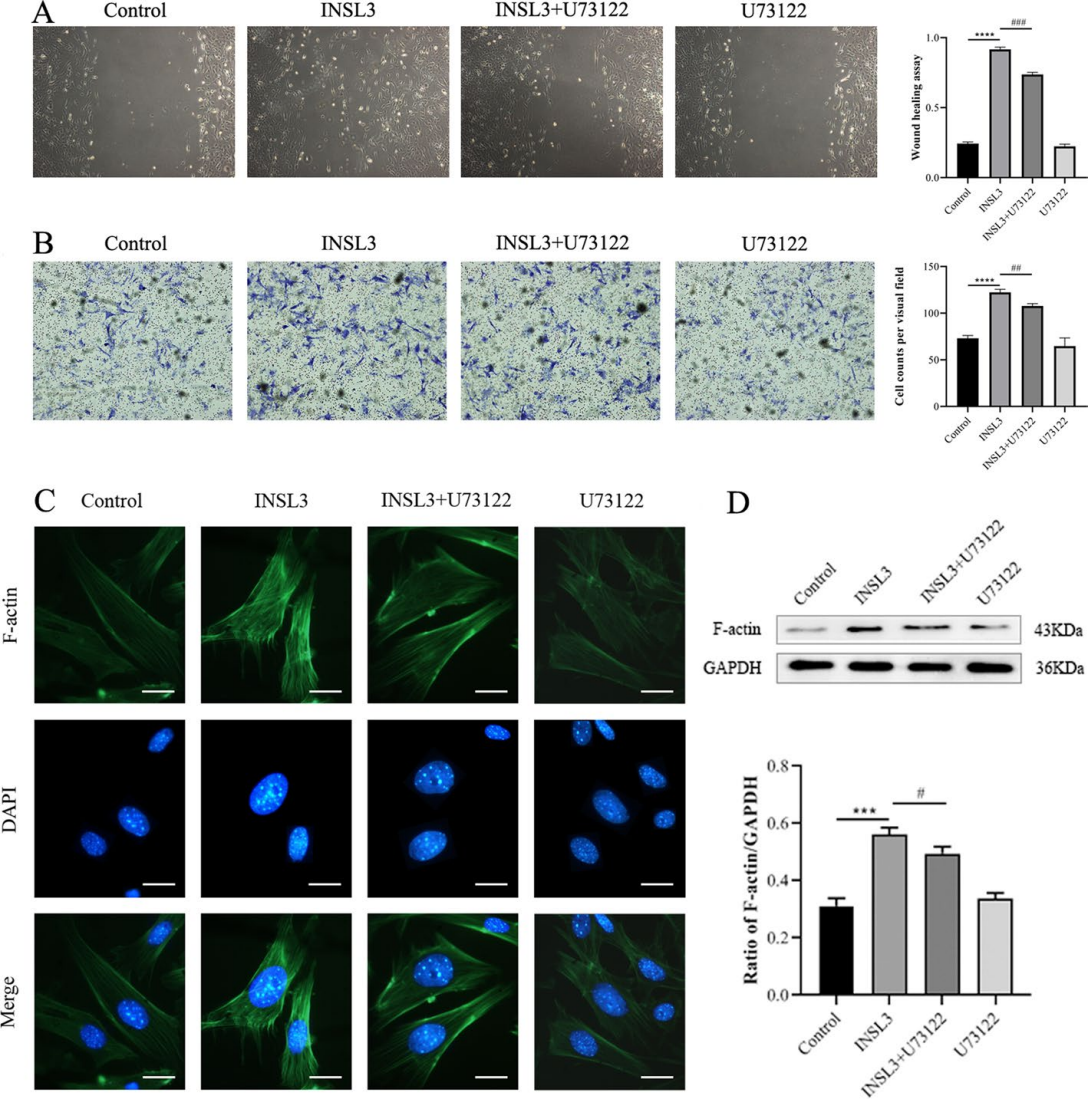
U73122 mediated the effect of INSL3 on the migration ability of gubernacular cells. **A, B** The scratch and transwell tests assessed gubernacular cell migration in each group. **C, D** The expression of fibrous actin (F-actin) protein was analyzed by immunofluorescence staining and Western blot. Scale bars, 20 μm. Data are mean ± standard deviation of three independent experiments. *^&#^P < 0.05, **^&##^P < 0.01, ***^&###^P < 0.001, and ****^&####^P < 0.0001

To assess the migration of gubernacular cells in each group, scratch assay and transwell assay tests were performed. The scratch assay results showed that the speed of wound healing was significantly better in the INSL3 group than in the control group, and wound healing was basically complete (P < 0.001); and the speed of wound healing was lower in the INSL3 + U73122 group than in the INSL3 group (P < 0.01) (Fig. 2A). The results of transwell experiment showed that the number of migrated cells was significantly higher in the INSL3 group than in the control group (P < 0.001). The number of migrated cells was less in the INSL3 + U73122 group than in the INSL3 group (P < 0.01) (Fig. 2B).

In addition, we determined the expression level of fibrous actin (F-actin) protein in gubernacular cells by immunofluorescence and western blot to further evaluate whether the migration of gubernacular cells was related to the contractile behavior. Immunofluorescence showed that in the control group, F-actin was mainly distributed in the periphery of the cells, forming peripheral actin ribbons, and a few short and fine stress fibers were observed in the cytoplasm. INSL3 stimulation caused cytoskeletal remodeling, increased actin ribbons around the cell, and made F-actin microfilaments in the cytoplasm significantly thicker and longer, most of which were thick stress filaments arranged along the cell edge; thus, suggesting that INSL3 induced cytoskeletal remodeling of gubernacular cells and increased the formation of stress fibers (Fig. 2C). Western blot showed that the expression of F-actin protein was increased in gubernacular cells of the INSL3 group compared with the control group (P < 0.001). The expression of F-actin protein was weaker in the INSL3 + U73122 group than in the INSL3 group (P < 0.05) (Fig. 2D).

These results indicated that INSL3 increased the migration ability of gubernacular cells, but U73122 inhibited the pro-migration effect of INSL3. F-actin-mediated contractile function may be involved in this process of cell movement.

### INSL3 reduced the apoptosis ability of gubernacular cells, and U73122 inhibited the anti-apoptosis effect of INSL3

**Fig. 3:**
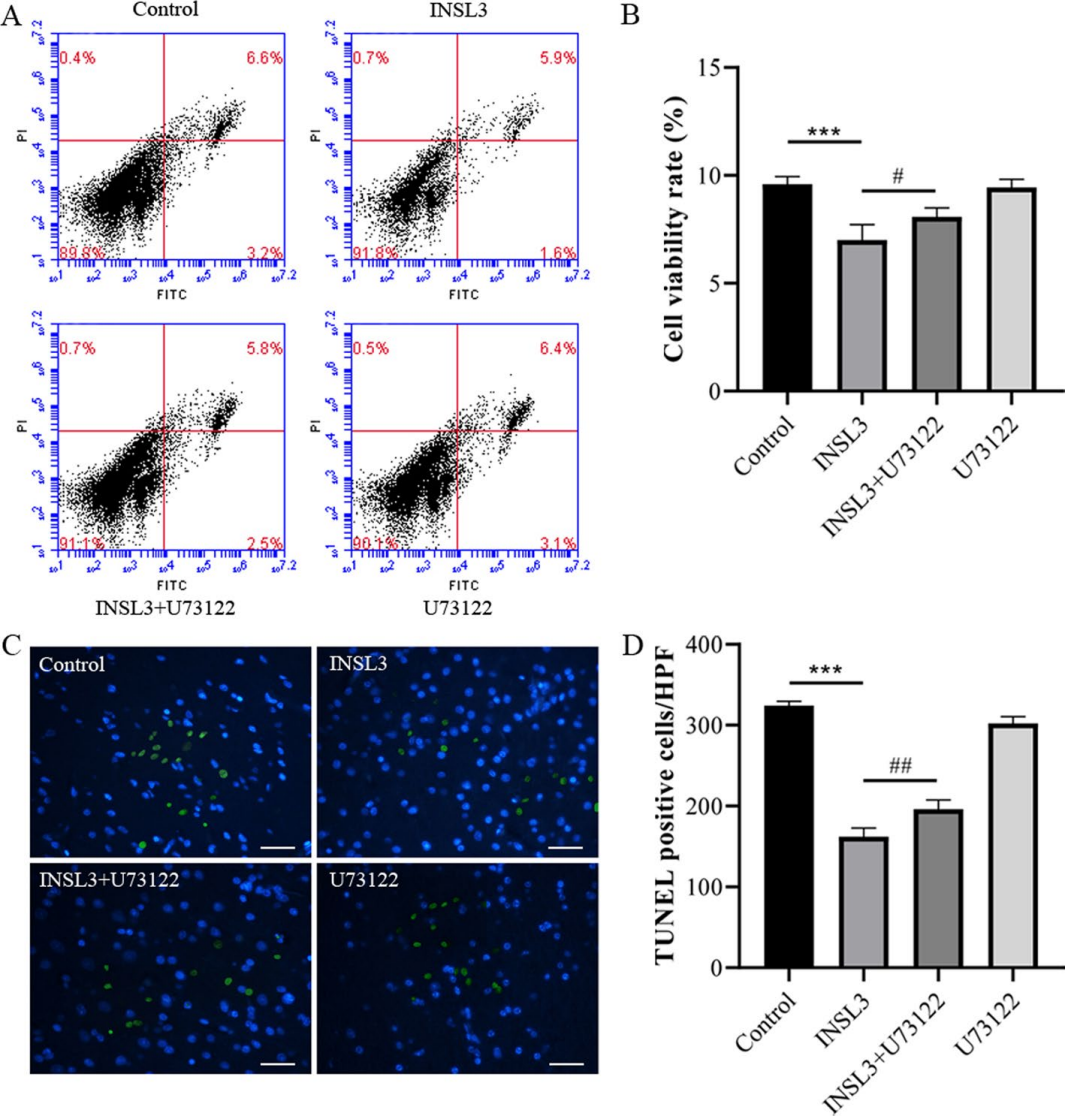
U73122 inhibited the anti-apoptotic effect of INSL3 on gubernacular cells. **A, B** Flow cytometry evaluate the effect of INSL3 on apoptosis of gubernacular cells. **C, D** Cell apoptosis was detected by TUNEL staining. Scale bars, 50 μm Data are mean ± standard deviation of three independent experiments. *^&#^P < 0.05, **^&##^P < 0.01, and ***^&###^P < 0.001

To evaluate the effect of INSL3 on apoptosis of gubernacular cells, flow cytometry and TUNEL staining were used to detect cell apoptosis. Flow cytometry results showed that compared with the control group, the apoptosis ability was significantly decreased in the INSL3 group (P < 0.001), while U73122 inhibited the anti-apoptotic effect of INSL3 (P < 0.01) (Fig. 3A, B). The effects of INSL3 and U73122 on apoptosis of gubernacular cells were verified again by TUNEL staining, and the results were consistent with the results of flow cytometry (Fig. 3C, D). These data suggested that INSL3 reduced the apoptosis ability of gubernacular cells, and U73122 inhibited the anti-apoptosis effect of INSL3.

### PLC/PKC activation was a novel signaling mechanism by which INSL3 regulated the biological characteristics of gubernacular cells

**Fig. 4:**
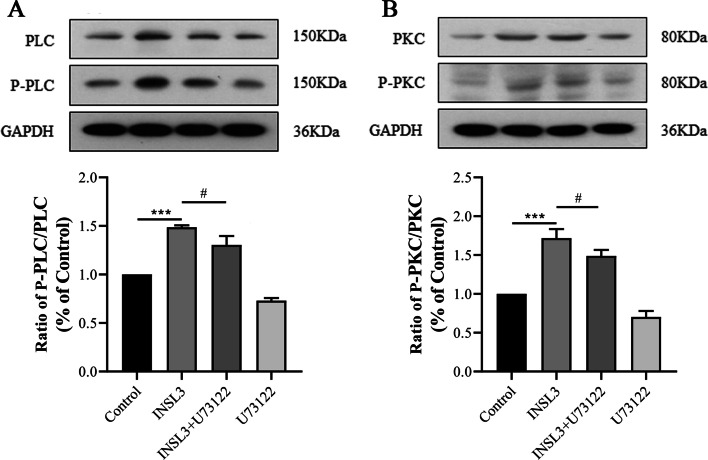
PLC/PKC was involved in the effect of INSL3 on gubernacular cells. **A** The change of P-PLC/PLC expression level was detected by western blotting. **B** The change of P-PKC/PKC expression level was detected by western blotting. Data are mean ± standard deviation of three independent experiments. *^&#^P < 0.05, and ***^&###^P < 0.001

In order to test whether PLC/PKC activation was a novel signaling mechanism by which INSL3 regulated the biological characteristics of gubernacular cells, we examined the effect of U73122 on the protein expression of active PLC and PKC in gubernacular cells. As shown in Fig. 4A, B, the protein levels of P-PLC/PLC and P-PKC/PKC were significantly increased in gubernacular cells after INSL3 administration compared with the control group (P < 0.001). INSL3 regulated the biological characteristics of gubernacular cells by enhancing PLC and PKC phosphorylation. However, pre-treatment with U73122 clearly reduced the phosphorylation of PLC/PKC protein induced by INSL3 (P < 0.05). These results suggested that INSL3 could affect the biological characteristics of gubernacular cells by activating the PLC/PKC pathway.

## Discussion

The important role of the gubernaculum in testicular descent has been confirmed in both human and animal studies [4, 22, 23]. In addition, it was observed that the change in the serum INSL3 concentration coincided with the testicular descent and the development of gubernaculum, and the testis completely descended into the scrotum during the increase in the INSL3 concentration [24, 25]. Mutations or polymorphisms in INSL3 and/or its specific receptor RXFP2 account for only a small proportion of cryptorchidism cases and cannot explain its pathogenesis in the majority of cases [26, 27]. Therefore, it is necessary to explore the mechanism by which INSL3 affects the gubernaculum. However, there are no reliable results on the mechanism by which INSL3 affects the gubernaculum through the receptor-signal transduction pathway.

Generally, GPCRs bind ligands and activate a series of signaling pathways in the cell, ultimately causing a change in the cell state [28, 29]. As a specific receptor for INSL3, RXFP2 is a GPCR on the membrane of the gubernaculum, which mediates a series of biological effects induced by INSL3 [30, 31]. In our previous study, it was found that DES could influence the rapid non-genetic effect on gubernacular cells by influencing a change in the intracytoplasmic free Ca^2+^ concentration, activating PLC, and then activating the PKC cell signaling pathway in gubernacular cells [19]. In the present study, to investigate whether INSL3 affected the biological characteristics of gubernacular cells through the RXFP2-PLC/PKC signaling pathway, the PLC inhibitor U-73122 was used to pretreat gubernacular cells and observe the effect of INSL3 on cell proliferation, migration, and apoptosis.

The results of our study showed that mouse gubernacular cells could be specifically labeled by the goat anti-mouse RXFP2 polyclonal antibody, which proved that RXFP2 was involved in the structure of the gubernacular cell membrane. INSL3 binds to the specific receptor RXFP2 located on the cell membrane to form the INSL3/RXFP2 complex, which then produces biological effects, stimulates the proliferation of the gubernaculum, and regulates the growth and development of the gubernaculum [8, 32, 33].

In this study, we found that INSL3 promoted the proliferation and migration of mouse gubernacular cells, but treatment with the PLC/PKC signaling pathway inhibitor U73122 significantly inhibited these effects of INSL3. The results of cell apoptosis detection showed that INSL3 had an anti-apoptotic effect on mouse gubernacular cells, and U73122 inhibited the anti-apoptotic effect of INSL3. Our results show that INSL3 can promote the proliferation and migration of gubernacular cells and inhibit their apoptosis, and the PLC/PKC signaling pathway is involved in these biological processes, which provides clear evidence that the PLC/PKC signaling pathway mediates the biological function of INSL3 in regulating the proliferation, migration and apoptosis of gubernacular cells.

To investigate the role of the PLC/PKC signaling pathway, we blocked PLC/PKC signaling pathway with U73122. The results showed that INSL3 significantly up-regulated PLC/PKC protein phosphorylation, while U73122 attenuated INSL3-induced PLC/PKC protein phosphorylation. Our results demonstrate that INSL3 regulates the regulation of proliferation, migration and apoptosis of mouse gubernacular cells by activating the PLC/PKC pathway.

It is well known that PCNA is a nuclear protein in the cell division phase, which plays a key role in DNA replication and cell cycle regulation. It only exists in proliferating cells, plays an important role in the initiation of cell proliferation, and is a good indicator of cell proliferation [34, 35]. In this study, we analyzed the expression of the PCNA protein using immunofluorescence staining and western blot, and we found that INSL3 could up-regulate the protein expression level of PCNA, while the PCNA expression was significantly weakened after U73122 pretreatment. These results suggest that the up-regulation of PCNA by INSL3 might be related to activation of the PLC/PKC signaling pathway. INSL3 promotes the proliferation of gubernacular cells by up-regulating PCNA expression through activating the PLC/PKC signaling pathway. The grading mechanism of INSL3 in promoting the proliferation of gubernacular cells was analyzed from a new perspective, which was not assessed in previous studies on the gubernaculum.

During the two stages of testicular descent, the migration of gubernacular cells is particularly active. Cell migration cannot be separated from cell contraction [36]. Cytoskeletal remodeling occurs during cell contraction, and F-actin plays an important role in cytoskeletal rearrangement [37–39]. The dynamic regulation of F-actin is the structural basis of many pathophysiological processes, including cell motility, division, morphogenesis, and intercellular protein transport [40]. The results of our study showed that the expression of F-actin protein was significantly up-regulated after INSL3 treatment. Meanwhile, the histological results showed that the peripheral actin ribbon was increased after INSL3 stimulation, and the microfilaments of F-actin in the cytoplasm were significantly thicker and longer, most of which were long thick stress filaments arranged along the edge of the cell. The increased F-actin level may promote cytoskeletal remodeling and increase stress fiber formation. These changes were significantly reduced by pretreatment with U73122. These data indicate that INSL3 regulates F-actin polymerization by activating the PLC/PKC signaling pathway, which mediates actin filament elongation to achieve skeletal reorganization and promote cell migration.

## Conclusion

**Fig. 5:**
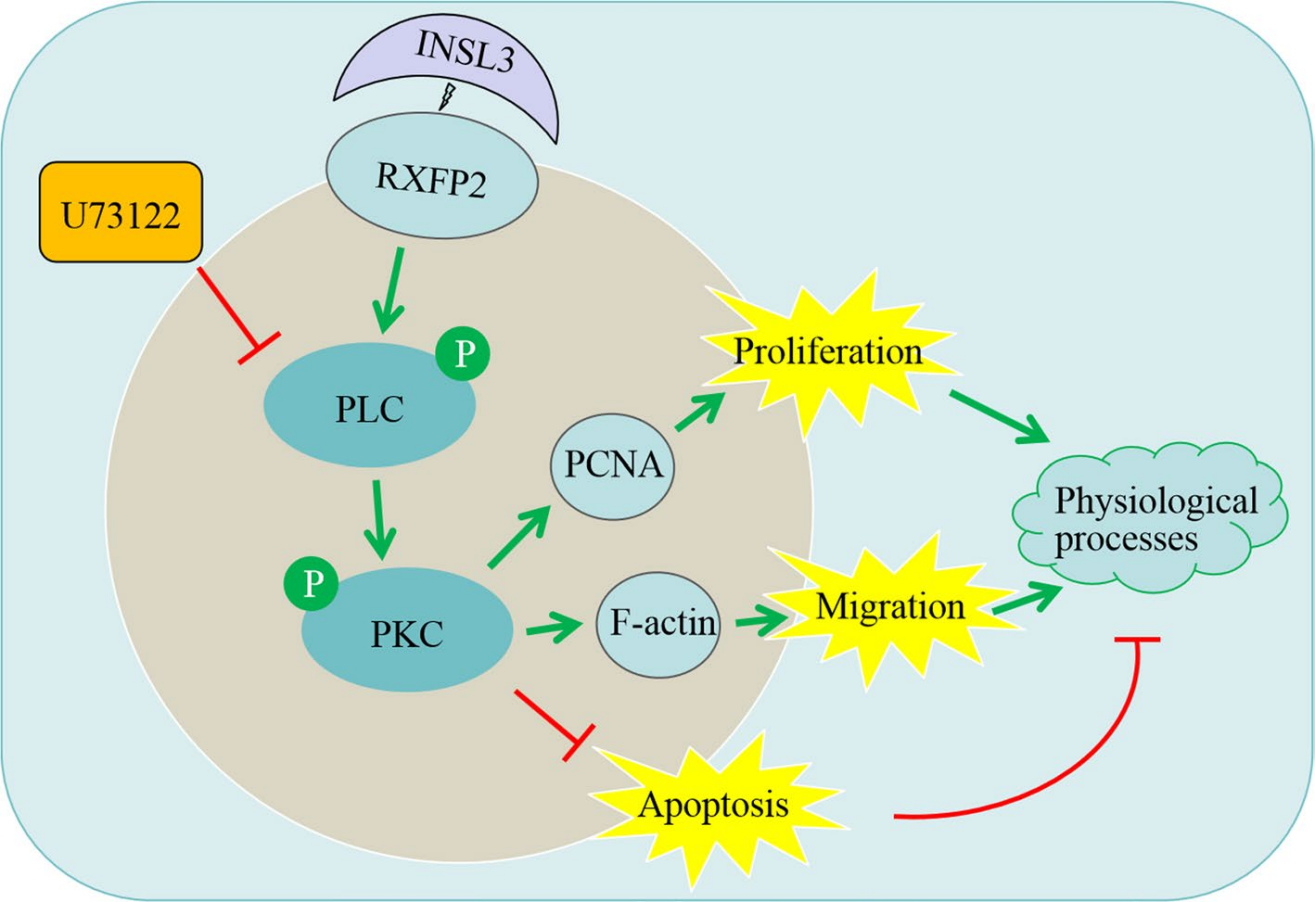
Graphic abstract. INSL3 regulated gubernacular cell proliferation, migration, and apoptosis via the PLC/PKC signaling pathway. Specifically, INSL3 binding to RXFP2 may up-regulate the expression levels of PCNA and F-actin by activating the PLC/PKC signaling pathway to promote the proliferation and migration of gubernacular cells and inhibit their apoptosis

In summary, our study demonstrated that INSL3 plays an important role in the growth of gubernacular cells, and it confirmed that INSL3 could promote the proliferation and migration of gubernacular cells and inhibit their apoptosis. Interestingly, U73122 inhibited the above regulatory effects of INSL3 on gubernacular cells. Further research firstly revealed that INSL3 binding to RXFP2 may up-regulate the expression levels of PCNA and F-actin by activating the PLC/PKC signaling pathway to promote the proliferation and migration of gubernacular cells (Fig. 5). These findings provide a novel insight into the molecular mechanisms by which INSL3 regulates growth of the gubernaculum.

## Supplementary Information

**Additional file 1: Figure S1.** A schematic diagram to illustrate the experimental system for function confirmation of INSL3 regulating gubernacular cells and signaling pathways analysis.

**Additional file 2: Figure S2.** The specific methods of cell counting.

**Additional file 3: Table S1.** The data of OD value of cell proliferation was detected by CCK-8.

## Data Availability

All data generated or analyzed during this study are included in this published article and its additional information files.

## Abbreviations

INSL3: Insulin like factor 3
RXFP2: Relaxin family peptide receptor 2
GPCR: G protein-coupled receptor
PLC: Phospholipase C
PKC: Protein kinase C
CCK-8: Cell counting kit-8
DES: Diethylbestrol
HE: Hematoxylin and eosin
DMEM: Dulbecco’s modified Eagle’s medium
FBS: Fetal bovine serum
FITC: Fluorescein isothiocyanate
PCNA: Proliferating cell nuclear antigen
