# Supplementary material for: The RXFP2-PLC/PKC signaling pathway mediates INSL3-induced regulation of the proliferation, migration and apoptosis of mouse gubernacular cells
Source: Cell Mol Biol Lett. 2023 Feb 27;28:16. doi: 10.1186/s11658-023-00433-0 (PMC9972740; doi:10.1186/s11658-023-00433-0)
Supplement: Supplementary file 1 — Additional file 1: Figure S1. A schematic diagram to illustrate the experimental system for function confirmation of INSL3 regulating gubernacular cells and signaling pathways analysis. [file 11658_2023_433_MOESM1_ESM.docx]

**Additional file 1**


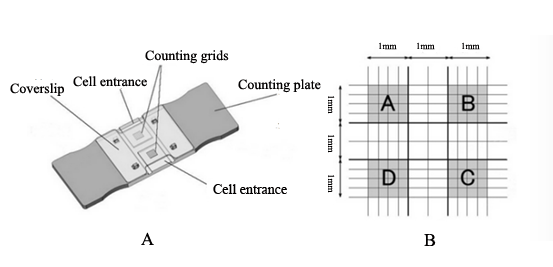


**Figure s1.** The cells in suspension were counted. A Cell counting plate, B Cell counting grids. A small amount of uniform cell suspension was dropped into the gap between the counting plate and cover slip from the cell entrance. Cells in the four regions were counted, and those on the line were counted only when they were in the left upper corner, not in the right lower corner. The cell concentration was calculated using the following equation: (number of cells/ml) = (total number of cells in four regions) × 10,000 × dilution fold.
