# Supplementary figures and images for: The RXFP2-PLC/PKC signaling pathway mediates INSL3-induced regulation of the proliferation, migration and apoptosis of mouse gubernacular cells
Source: Cell Mol Biol Lett. 2023 Feb 27;28:16. doi: 10.1186/s11658-023-00433-0 (PMC9972740; doi:10.1186/s11658-023-00433-0)

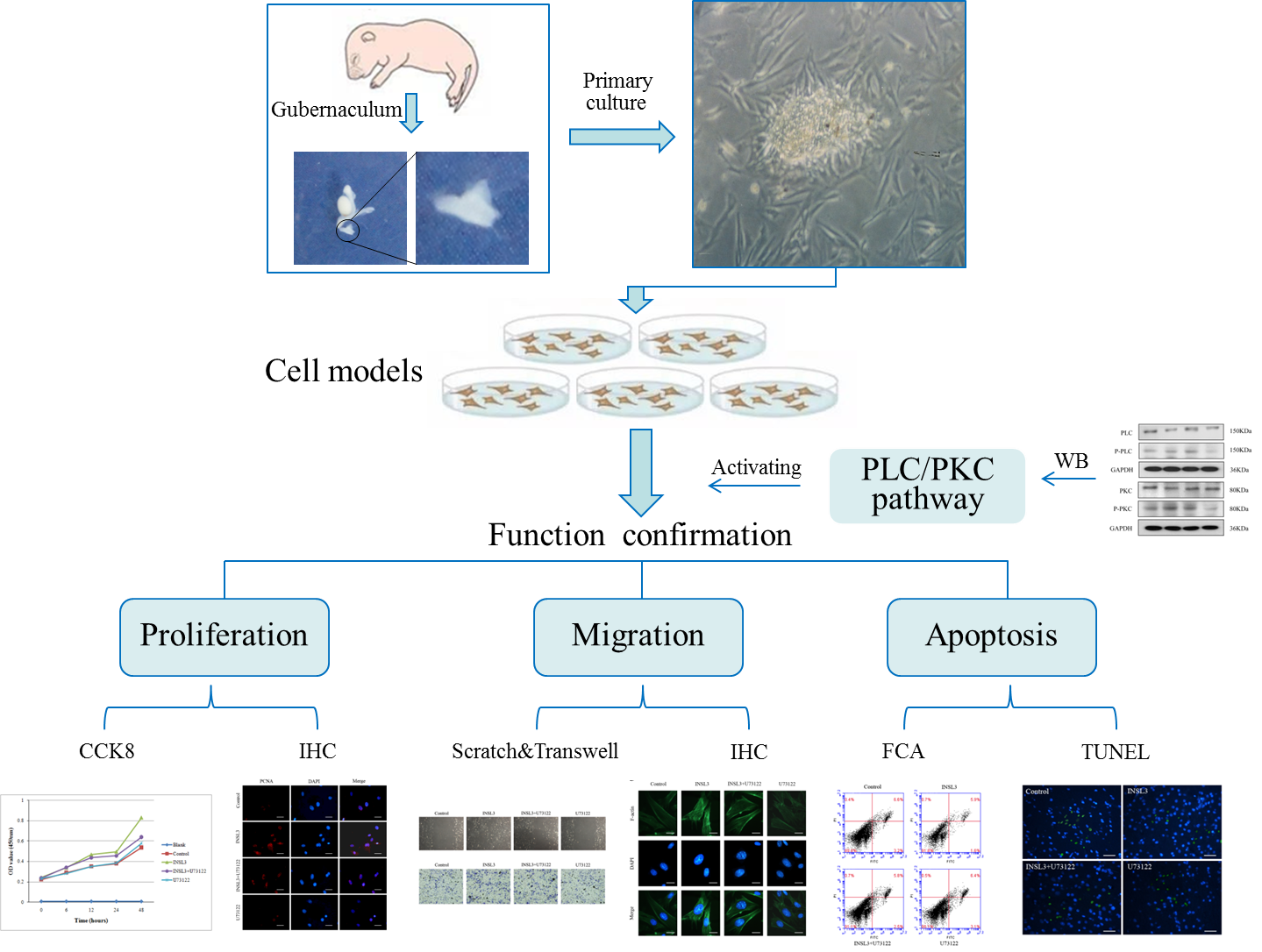

Supplement: Supplementary file 2 — Additional file 2: Figure S2. The specific methods of cell counting. [file 11658_2023_433_MOESM2_ESM.tif]
