# Supplementary material for: The RXFP2-PLC/PKC signaling pathway mediates INSL3-induced regulation of the proliferation, migration and apoptosis of mouse gubernacular cells
Source: Cell Mol Biol Lett. 2023 Feb 27;28:16. doi: 10.1186/s11658-023-00433-0 (PMC9972740; doi:10.1186/s11658-023-00433-0)
Supplement: Supplementary file 3 — Additional file 3: Table S1. The data of OD value of cell proliferation was detected by CCK-8. [file 11658_2023_433_MOESM3_ESM.docx]

**Additional file 3**

**Table S1.** The OD value of cell proliferation was detected by CCK-8 (‾X±S ).

|  | 0h | 6h | 12h | 24h | 48h |
| --- | --- | --- | --- | --- | --- |
| Blank | 0.009±0.0001 | 0.009±0.0005 | 0.009±0.0005 | 0.009±0.0006 | 0.008±0.0002 |
| Control | 0.223±0.0074 | 0.291±0.0231 | 0.351±0.0314 | 0.378±0.0314 | 0.537±0.0221 |
| INSL3 | 0.244±0.0305 | 0.340±0.0238 | 0.468±0.0083 | 0.496±0.0083 | 0.830±0.0711 |
| INSL3+U73122 | 0.236±0.0225 | 0.342±0.0196 | 0.437±0.0224 | 0.455±0.0224 | 0.639±0.0507 |
| U73122 | 0.231±0.0161 | 0.283±0.0225 | 0.349±0.0225 | 0.385±0.0265 | 0.580±0.0316 |
